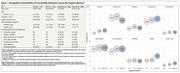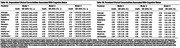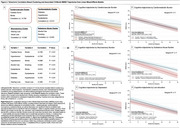# Multimorbidity and Cognitive Trajectories in Resource‐Limited Settings: Real‐World Evidence from a Brazilian Public Memory Clinic

**DOI:** 10.1002/alz70860_101604

**Published:** 2025-12-23

**Authors:** Fernando Jacob Lazzaretti, Alessandra Santini, Álvaro De Leonço, Andrei Bieger, Andressa de Oliveira Felício, Barbara Suman Bahlis, Cristiano Aguzzoli, Eduarda Faresin, Fabricio Nery Garrafiel, Julia Patatt, Lorenza P. Botton, Maria Lúcia Hristonof, Maria Rosa Alves da Silva, Lucas Porcello Schilling

**Affiliations:** ^1^ School of Medicine, Pontifícia Universidade Católica do Rio Grande do Sul (PUCRS), Porto Alegre, Rio Grande do Sul, Brazil; ^2^ Psychiatry Department, São Lucas Hospital of PUCRS, Porto Alegre, Rio Grande do Sul, Brazil; ^3^ Universidade Federal do Rio Grande do Sul, Porto Alegre, Rio Grande do Sul, Brazil; ^4^ Neurology Department, São Lucas Hospital of PUCRS, Porto Alegre, Rio Grande do Sul, Brazil; ^5^ Global Brain Health Institute (GBHI), San Francisco, CA, USA; ^6^ Brain Institute of Rio Grande do Sul (InsCer), Porto Alegre, Rio Grande do Sul, Brazil; ^7^ Pontifícia Universidade Católica do Rio Grande do Sul (PUCRS), Porto Alegre, Rio Grande do Sul, Brazil

## Abstract

**Background:**

Dementia often co‐occurs with multiple comorbidities that may accelerate cognitive decline. Low‐ and middle‐income countries (LMICs) face a disproportionate dementia burden due to resource limitations, elevated comorbidity prevalence, and rapid population aging. However, most comorbidity data are derived from well‐characterized cohorts, underscoring the need for real‐world LMIC evidence. We aimed to identify clinical comorbidities associated with dementia and assess how comorbidity clusters influence longitudinal cognitive decline.

**Method:**

We retrospectively analyzed 500 adults who were at least once evaluated over the past 10 years at a tertiary neurology memory clinic within the Brazilian Unified Healthcare System. Participants were classified as subjective cognitive decline (SCD), mild cognitive impairment (MCI), or dementia (due to Alzheimer's disease, vascular, or other subtypes). One‐way ANOVA and Fisher's exact test assessed baseline demographic characteristics. Unpenalized and ridge‐penalized logistic regressions assessed the associations between 10 binary comorbidities and dementia, adjusting for age and sex. Tetrachoric correlation–based clusters informed longitudinal linear mixed‐effects analyses of MMSE trajectories.

**Result:**

Dementia participants were older (74±10.3 years) and had lower educational attainment (3.1±3.7 years). Comorbidity prevalence was high across groups, with hypertension and low education exceeding 65% of participants (Figure 1B). Unpenalized logistic models identified stroke (OR=3.53; 95% CI, 1.65–7.57) and alcohol use (OR=6.56; 95% CI, 1.69–25.43) as significant predictors of dementia, and stroke remained significant in sensitivity analyses excluding vascular dementia cases (Models 1 and 2, Table 1A). Ridge‐penalized regressions, applied to address high correlations among variables, corroborated these findings with slightly attenuated effect sizes (Table 1B). Cluster analyses revealed four comorbidity groups (cerebrovascular, cardiometabolic, neurosensory, substance abuse). Longitudinally, higher cerebrovascular burden at baseline was linked to a faster annual MMSE decline (−0.43 points/month; *p* <0.01), whereas neurosensory and substance abuse burden also trended toward steeper decline (Figure 2).

**Conclusion:**

In this real‐world LMIC sample, multimorbidity was pervasive, and cerebrovascular, neurosensory and substance abuse burdens emerged as significant contributors to dementia risk and accelerated cognitive decline. Low education, common in LMICs, further amplified brain health vulnerability. Findings underscore the need for interventions at primary care levels to mitigate progression and reduce the overall dementia burden in resource‐limited settings.